# Supplementary figures and images for: Coupling and uncoupling of midline morphogenesis and cell flow in amniote gastrulation
Source: eLife. 2024 May 10;12:RP89948. doi: 10.7554/eLife.89948 (PMC11087055; doi:10.7554/eLife.89948)

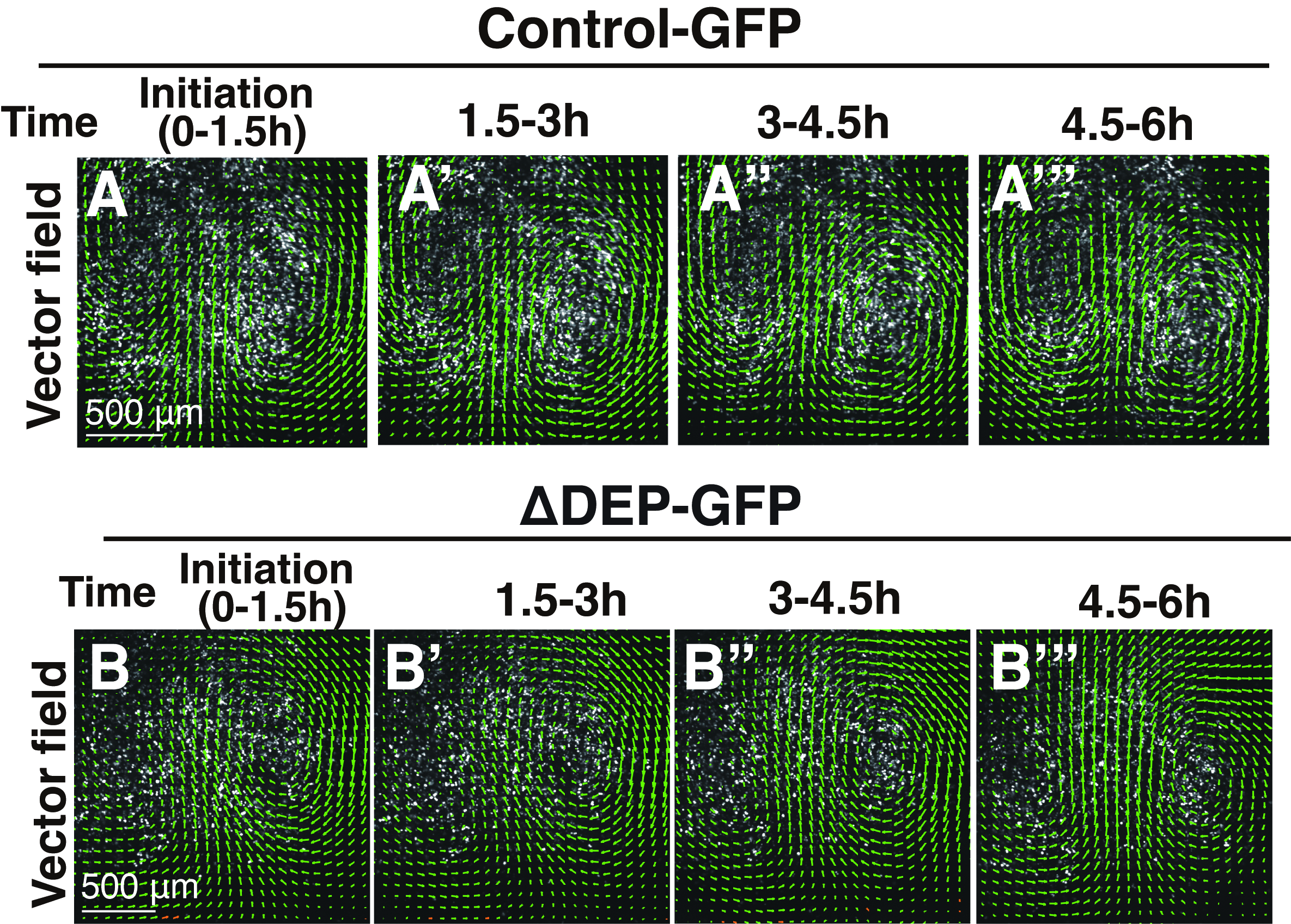

Supplement: Figure 1—source data 1. [file elife-89948-fig1-data1.tif]

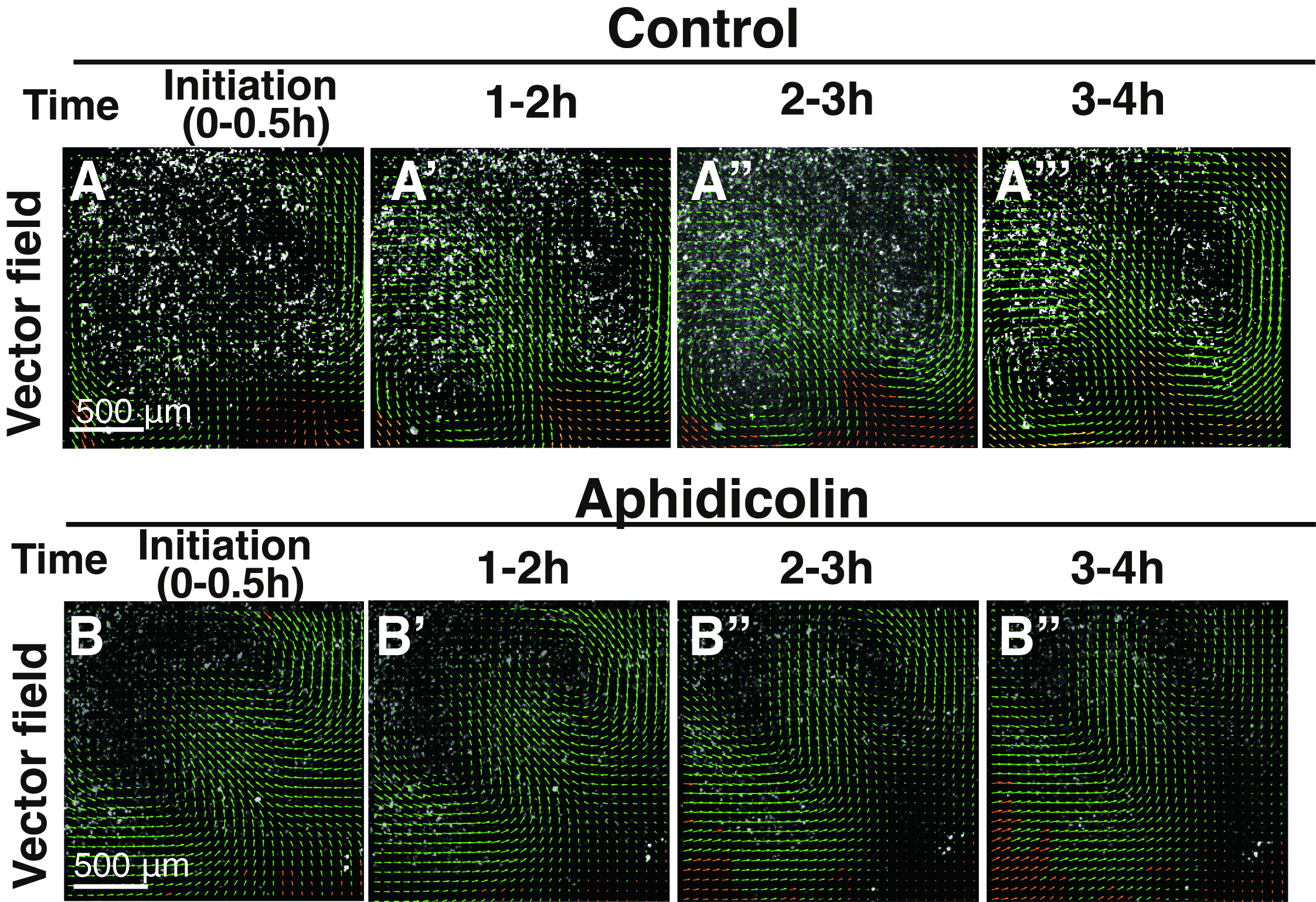

Supplement: Figure 2—source data 1. [file elife-89948-fig2-data1.tif]

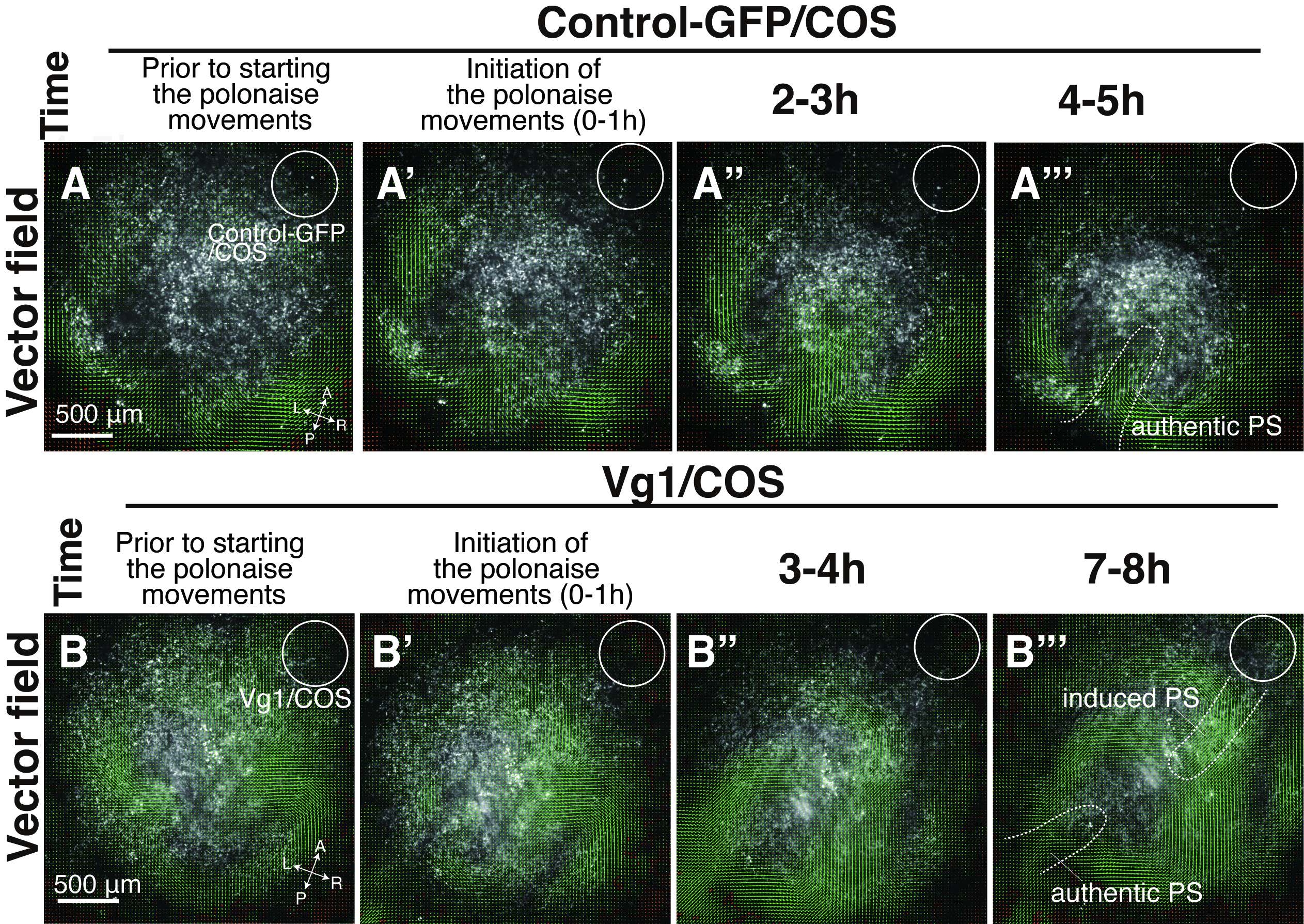

Supplement: Figure 3—source data 1. [file elife-89948-fig3-data1.tif]

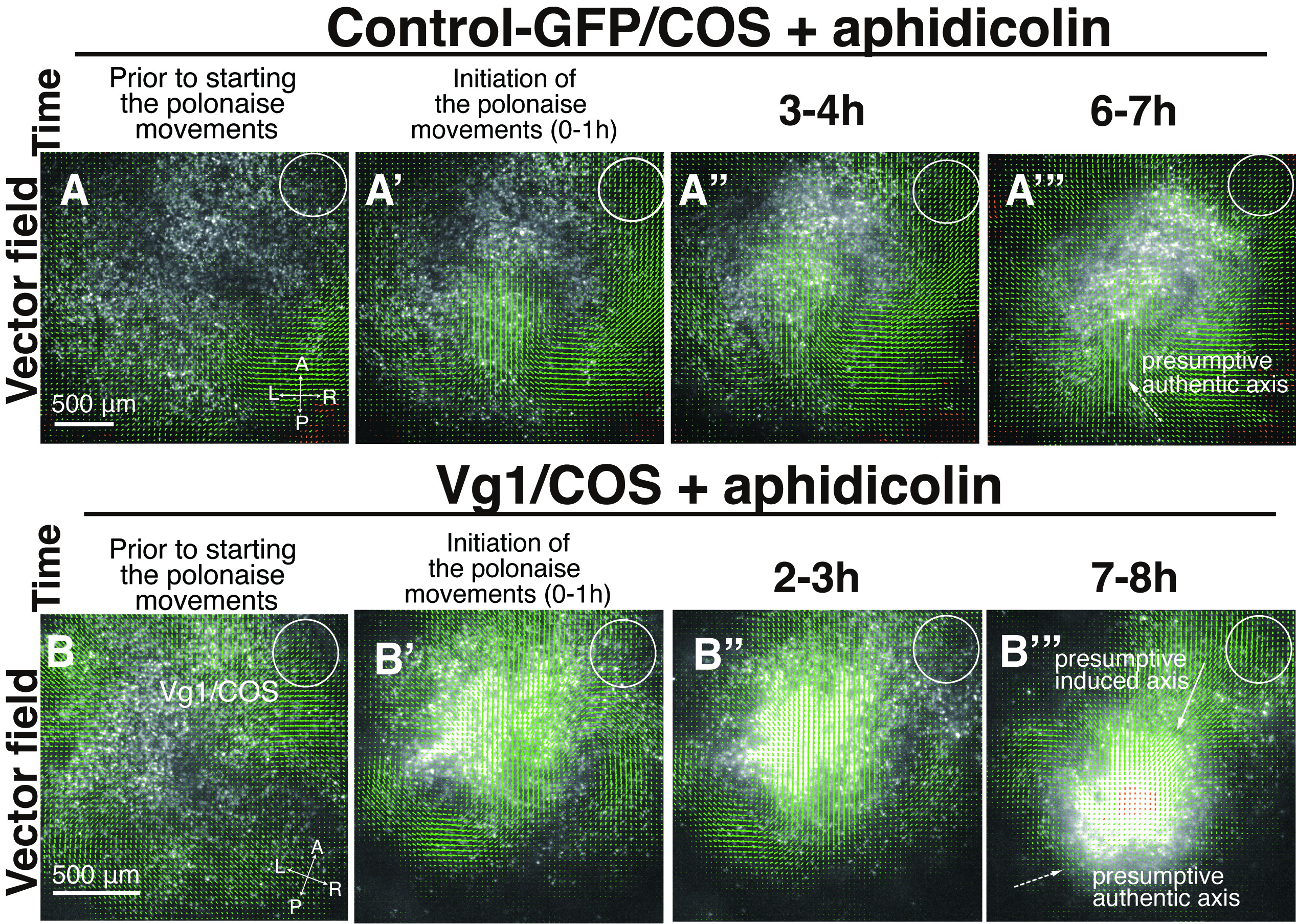

Supplement: Figure 4—source data 1. [file elife-89948-fig4-data1.tif]
